# The evolution and driving mechanism of education inequality in China: From 2003 to 2020

**DOI:** 10.1371/journal.pone.0314297

**Published:** 2025-01-31

**Authors:** Yuanzhi Guo, Xuhong Li

**Affiliations:** 1 Institute of Geographic Sciences and Natural Resources Research, Chinese Academy of Sciences, Beijing, P.R.China; 2 Key Laboratory of Regional Sustainable Development Modelling, Chinese Academy of Sciences, Beijing, P.R.China; 3 University of Chinese Academy of Sciences, Beijing, P.R.China; National Institute of Demographic Studies: INED, FRANCE

## Abstract

Although China’s education development has made great progress, there are obvious regional differences in China’s educational development. A systematic investigation of the regional inequality in China’s educational development and its driving factors is of great significance for optimizing the allocation of educational resources and giving full play to the critical role of education in regional development. In addition, the research on the evolution and internal mechanism of educational development inequality in China can also provide experience and reference for the Global South. Therefore, we construct a comprehensive evaluation index system to measure the level of regional educational development, reveal the regional inequalities in China’s educational development, and employ spatial econometric model to dissect the factors influencing the regional inequalities. The results show that China’s educational development level continues to increasing from 2003 to 2020, but a significant decrease in its growth rate. In this process, regional differences in education inequality in China have gradually narrowed, which can be confirmed by changes in the Gini coefficient and Theil index. In terms of direct spillover effects, the per capita fiscal expenditure on education and urbanization rate have positive effects. In terms of indirect spillover effects, per capita GDP and per capita fiscal expenditure on education have negative effects, while population density and urbanization rate have positive effects. After replacing the weight matrix and removing the extreme values, the model also passes the robustness test. However, this mechanism is heterogeneous in different regions, therefore, we put forward the corresponding policies and measures according to the regional driving effects of influencing factors.

## 1. Introduction

Education is a human activity that transmits social values and accumulated knowledge [1]. Through the accumulation of human capital, education promotes the personalization and socialization of individuals and boosts regional development to a higher level. Because of the critical role of education in regional development [2, 3], both developing and developed countries have placed great emphasis on educational development [4, 5], and have made great efforts to ensure equal access to education for all [6]. Meanwhile, the connotation of educational development is being deepened and expanded to meet the needs of the transformation and upgrading of people’s demand. The Millennium Development Goals (MDGs), introduced in 2000, focus on universal primary education in the field of education, i.e., completion of primary education for both boys and girls [7]. The Sustainable Development Goals (SDGs) issued in 2015 focus on quality education, that is, ensuring inclusive and equitable quality education and promoting lifelong learning opportunities for all by 2030 [8].

In China, education is widely valued and the government has given priority to educational development [9]. Since the founding of the People’s Republic of China (PRC) in 1949, the Chinese government has continued to meet people’s growing demand for multi-level and diversified education by increasing the supply of educational resources and improving school conditions. On the other hand, China has also improved its policy system to ensure equity in education. From 1964 to 2020, China’s illiteracy rate decreased from 33.58% to 2.67%, and the average years of schooling for people aged 15 and above increased from 2.22 to 9.91 years [10]. Currently, China has built the world’s largest educational system and has basically realized the transformation from a country with a large population to a country with a large human capital [11]. Through effects such as the accumulation of human capital and the improvement of production efficiency, education has become an important driving force for national economic and social development [12]. According to Zhu et al. (2008), education contributed 6.7% of China’s economic growth in the period of 1999–2003, and the contribution rate of education in the eastern region was significantly greater than that in the central and western regions [13]. Jamison et al. (2010) also found that the contribution rate of education to China’s economic growth ranged from 5% to 8% in 1970–2000 [14]. In the context of demographic transition, the increase in educational attainment has effectively improved the quality of the working-age population, mitigating the negative impact on China’s economic growth due to the disappearance of the demographic dividend caused by below-replacement fertility and population aging [15].

Currently, China’s economic development is in a critical period of transformation and upgrading [16, 17]. There is an urgent need to transform the economic growth model from factor- and investment-driven to technology- and innovation-driven [18]. To achieve this goal, the key is to cultivate talents and the foundation is to develop education. due to the regional differences in human and natural conditions, there are obvious disparities in China’s educational development. However, there are obvious regional development disparities in China, which leads to the unequal development of education. Regional educational inequality refers to the phenomenon that there are significant differences in educational resources, educational opportunities, educational quality and educational achievements among regions, which damages educational equity. In 2023, the per capita GDP of eastern China will reach 102,700 yuan per person, while that of central China and western China will be 73,100 yuan and 67,100 yuan respectively. In addition, the eastern region is significantly higher than the central and western regions in terms of social security, public service supply and resident income. Although the right to education and the right to access to education opportunities are guaranteed in China through legislative means and the provision of subsidies for compulsory education, there are still large differences between regions in terms of advanced educational hardware and software facilities and quality teachers, and promoting the balanced development of education has become an important issue in Chinese society. Besides, China has a population of more than 1.4 billion people, the huge population size leads to the complexity of its education development. In addition, as the world’s largest developing country, China’s educational development was also relatively backward. According to the World Bank, the preprimary enrolment rate in China and Kenya in 2003 was 44.86% and 40.76%, respectively, but in 2019, that data reached 89.12% and 65.14%, respectively. The rapid educational development in China can provide a reference for the Global South, and its emerging inequality can also serve as an early warning for these countries.

Therefore, analyzing regional inequality in China’s educational development and its driving factors is of great significance to make up for the shortcomings in education and provide a path to equal development of education for China and the Global South. Employing China’s education statistics from 2003 to 2020, this study constructs a comprehensive evaluation index system to measure the educational development index (EDI), reveals regional inequalities in China’s educational development, and uses a spatial panel model to analyze the factors influencing regional differences in China’s educational development. In line with the new situation and problems faced by China’s social and economic development, targeted measures are proposed to promote the high-quality development of education.

The rest of the article is organized as follows. Section 2 briefly reviews the relevant literature. Section 3 introduces the material, constructs an index system to evaluate the level of regional educational development, and defines the potential factors influencing the uneven development of regional education along with modelling the relationship between them. Section 4 presents the results, while Section 5 provides discussions on the findings and highlights the potential implications for policy making in the context of social and economic transformation. The study is finally concluded in Section 6.

## 2. Literature review

Before the reform and opening-up in 1978, the government assumed the primary responsibility for educational provision and funding, and provided a large number of educational opportunities for the children of workers and peasants, which transformed China’s educational development from a state of great inequality to one of relative equality [19, 20]. After that, selecting and training talents for economic development replaced eliminating class differences as the main function of education [19]. In this context, the Chinese government actively promotes educational reform, decentralizes administration and finance, and privatizes educational costs [20–22]. Although these measures have mobilized new resources to support educational development, they have also exacerbated regional educational inequality [22].

With the market mechanism replacing the government’s leading role in resource allocation, educational resources tend to gather in geographically advantageous areas, leading to regional inequalities in education, which are mainly manifested in urban-rural inequalities [6, 23–26], coast-inland inequalities [6, 20], as well as inequalities between underdeveloped and developed areas [26, 27]. Driven by social and economic development, urban-rural inequality has gradually replaced coast-inland inequality as the main feature of regional educational inequality [6]. Through affecting the accumulation of human capital, educational inequality also plays an important role in economic development and is a significant cause of regional economic disparities [28, 29]. The existing research shows that differences in knowledge capital account for 20%-30% of the state variation in per capita GDP in US [30]. A century of evidence shows that there is a significant positive correlation between income inequality and inequality in access to university education resources in US [4], and optimizing the allocation mechanism of university admission places can promote social integration and promote intergenerational mobility [31]. These studies provide useful reference and enlightenment for the balanced development of Chinese education.

Spatially, the extent of its impact varies by regions. In developed areas, the impact of educational inequality on economic development is limited; while its negative impact is significant in backward areas [32]. In addition, educational inequality poses a series of problems and challenges to regional development, including social fragmentation and political instability, which is detrimental to regional sustainable development and contrary to the aims of the Chinese government [19, 25, 33]. As people pay more attention to children’s education, educational inequality has also become one of the key factors driving population migration [34].

Years of schooling is one of the most used indicators to analyze regional inequalities in China’s educational development [6, 24–26]. In addition, indicators such as enrollment rate [23, 25], academic performance [35], educational expenditure [22, 36], and educational continuation opportunities [37] are also frequently used. Some studies examined the degree of regional inequality in educational development by calculating the standard deviation, the Gini coefficient, and the Theil index of these indicators [6, 26]. In terms of data sources, these studies generally use national census data [22, 26] as well as sample survey data, such as the Chinese General Social Survey (CGSS) [25], the China Family Panel Studies Survey (CFPS) [38, 39], and the China Health and Nutrition Survey (CHNS) [22]. Besides, the OECD’s Program of International Student Assessment (PISA) also provides important data to understand regional educational inequalities in China [40]. However, it should be noted that census data are not continuous and there are often biases between sample survey data and the true picture.

China’s vast size determines the roots of regional inequality in educational development. Since the distribution of educational resources is the result of human activity, natural conditions play a fundamental role in regional educational inequality [41]. In the context of educational decentralization and marketization of education services, economic disparities are the direct cause of regional educational inequality [20, 22, 42]. Irrational policy design is the institutional cause of regional educational inequality [43, 44], especially the urban-rural inequality in educational development. Except for the urban-rural differences in physical geography and socio-economic development, the urban-rural dual structure based on household registration (*hukou*) system plays a decisive role in urban-rural educational inequality [24–26, 45, 46]. Besides, factors such as regional culture [47] and urban development [37, 48] are also important causes of regional educational inequality. At the micro level, demographic characteristics such as nationality and occupation, as well as family background such as income and parental education, affect regional inequalities in educational development by influencing individual/family decision-making [36, 37].

Regional inequality in educational development is a complex socio-economic phenomenon [49]. However, most of the existing studies employ a single indicator to investigate this phenomenon and lack a comprehensive understanding of this phenomenon; the analysis of the causes of regional educational inequality is also conducted mainly at the micro level. Therefore, the contribution of this study is twofold. Firstly, the connotation of educational development is explored, and an index system is constructed to measure the level of regional educational development in two dimensions: educational opportunity and educational achievement. The former includes three sub-dimensions of primary school, junior secondary school, and senior secondary school; and the latter includes two sub-dimensions of educational attainment and enrollment rate. Secondly, based on revealing the characteristics of spatial inequality in China’s educational development, the spatial panel model is employed to investigate the factors influencing the spatial differences in educational development, and countermeasures for the high-quality development of China’s regional education are proposed.

## 3. Materials and methods

### 3.1. Index system and evaluation

Regional inequality in educational development refers to the disparity in educational development between regions. According to [50], educational development consists of three components, i.e., educational right, educational opportunity, and educational achievement. Since the Chinese government has legislated to guarantee equal rights in education [51], this study focuses on the latter two aspects of educational opportunity and educational achievement to construct a comprehensive evaluation index system to measure EDI. The former refers to the way in which individuals can acquire certain skills and knowledge, while the latter is about what happens to the population because of educational objectives and outputs. Based on the principles of data availability and systematicity, and with reference to relevant studies [22, 36, 41, 48], a comprehensive evaluation index system including 2 dimensions, 5 sub-dimensions and 26 indicators is constructed to measure the level of educational development and analyze the determinants of regional inequality in China’s educational development (Table 1).

**Table 1:** Comprehensive evaluation index system of EDI.

Dimension	Subdimension	Indicator	Effect	Weight
Educational opportunity	Primary school	Teacher-student ratio (-)	Positive	0.0368
		Class size (Students)	Negative	0.0359
		Number of books per student in the library (Volumes)	Positive	0.0376
		Number of computers per student (Sets)	Positive	0.0324
		Value of fixed assets per student (10,000 RMB)	Positive	0.0248
		Ratio of full-time teachers with bachelor’s degree or above (%)	Positive	0.0457
		Ratio of full-time teachers with middle-level or above titles (%)	Positive	0.0359
		Ratio of external teaching sites to the number of primary schools (%)	Negative	0.0387
	Junior secondary school	Teacher-student ratio (-)	Positive	0.0378
		Class size (Person)	Negative	0.0446
		Number of books per student in the library (Volumes)	Positive	0.0365
		Number of computers per student (Sets)	Positive	0.0416
		Value of fixed assets per student (10,000 RMB)	Positive	0.0448
		Ratio of full-time teachers with bachelor’s degree or above (%)	Positive	0.0320
		Ratio of full-time teachers with middle-level or above titles (%)	Positive	0.0379
	Senior secondary school	Teacher-student ratio (-)	Positive	0.0356
		Class size (Person)	Negative	0.0383
		Number of books per student in the library (Volumes)	Positive	0.0415
		Number of computers per student (Sets)	Positive	0.0263
		Value of fixed assets per student (10,000 RMB)	Positive	0.0396
		Ratio of full-time teachers with bachelor’s degree or above (%)	Positive	0.0275
		Ratio of full-time teachers with middle-level or above titles (%)	Positive	0.0437
Educational achievement	Educational attainment	Ratio of population with education level of junior secondary schools and above in population aged 6 and above (%)	Positive	0.0504
	Enrollment rate	Ratio of primary school graduates entering junior secondary schools (%)	Positive	0.0364
		Ratio of junior high school graduates entering senior secondary schools (%)	Positive	0.0472
		College entrance examination admission rate of “985” university (%)	Positive	0.0505

In terms of educational opportunity, we select teacher-student ratio and class size to reflect the degree of teacher guidance that each student can receive. Number of books per student in the library, number of computers per student and value of fixed assets per student are selected to represent the school physical education resources supply level. The level of teachers is an important aspect of educational development, thus we include ratio of full-time teachers with bachelor’s degree or above and ratio of full-time teachers with middle-level or above titles into the comprehensive evaluation index. School is the most important place for students to obtain education, and when there are abundant educational resources outside the school, it may increase educational inequality. Therefore, we select ratio of external teaching sites to the number of primary schools as a negative indictor. In terms of educational achievement, we select indicators from educational attainment and enrolment rate, including ratio of population with education level of junior secondary schools and above in population aged 6 and above, ratio of primary school graduates entering junior secondary schools, ratio of junior high school graduates entering senior secondary schools and college entrance examination admission rate of “985” university.

Since the entropy weighting method only considers the degree of data dispersion when determining the indicator weights [52]. Its weight design is not affected by subjective will, so it is widely used in the calculation of composite index [53]. Here, an improved entropy weighting method, which integrates the analytical hierarchy process (AHP) and entropy weighting method, is employed to determine the weights of each indicator [54, 55]. The calculation steps are as follows:

(1) The min-max normalization is used to normalize *x*_*ij*_, which is the value of indicator *j* in province *i*. Formulas for the normalization of positive and negative indicators are as follows, respectively:

$$y_{ij}=\left(x_{ij}-\min(x_{j})\right)/\left(\max(x_{j})-\min(x_{j})\right)$$ (1)

$$y_{ij}=\left(\max(x_{j})-x_{ij}\right)/\left(\max(x_{j})-\min(x_{j})\right)$$ (2)

(2) The entropy of indicator *j* is defined as follows:

$$e_{j}=\frac{-\sum_{i=1}^{m}p_{ij}\;\ln\;p_{ij}}{\ln\;m}$$ (3)

Where *m* is the total number of provinces, *p*_*ij*_ln*p*_*ij*_ is set as zero if *p*_*ij*_ is equal to zero and

$$p_{ij}=y_{ij}/\sum_{i=1}^{m}y_{ij}\;I=1,2,\ldots ,m;j=1,2,\ldots ,n$$ (4)

(3) The deviation of indicator *j* is calculated as follows:

$$D_{j}=1-e_{j}$$ (5)

A large value of *D*_*j*_ indicates a larger variation of indicator *j*, i.e., the greater the influence of indicator *j*.

(4) By using the AHP method, the subjective weight $\omega_{j}^{\prime }$ of each indicator is calculated. Please refer to [56] for detailed steps. Then, the final weight *ω*_*j*_ is calculated:

$$\omega_{j}=\omega_{j}^{\prime }D_{j}/\sum_{j=1}^{n}\omega_{j}^{\prime }D_{j}$$ (6)

In line with the normalized values and the weights of each indicator, the EDI of province *i* is calculated:

$$EDI_{i}=\sum_{i=1}^{n}\omega_{j}y_{ij}$$ (7)

### 3.2. Variable selection and model construction

Education is a public social activity [57], and its development level in a specific region depends on the structural relationship between the supply of educational resources and the demand for educational services. The NEG approach, proposed by Krugman (199l), aims to explain the uneven distribution of economic activity across space driven by factor migration governed by interplay of agglomeration and dispersion forces [58, 59]. Therefore, when exploring the regional inequality of education, we draw on this theory to select the factors that may lead to the flow of factors, and then reveal their impact on educational inequality. Meanwhile, due to the geospatial barriers, there is an accessibility between educational supply and demand [60]. Therefore, this study pre-selects six variables from the three dimensions of supply, demand and accessibility to examine the factors influencing regional inequality in China’s educational development (Table 2). First, the supply of educational resources reflects the educational opportunities provided to the school-age population by various market players such as the government and enterprises, and is closely related to government policies and regional economic development [61]). Here, per capita GDP is chosen to represent the level of regional economic development; per capita fiscal expenditure on education is used to characterize government policy support for education. Second, the demand for educational services refers to the educational needs that society and individuals can afford, including two aspects of quantity and level. The former depends on population size, while the latter is closely related to living standards. Here, population density and urbanization rate are employed to feature population size and living standard, respectively. Third, the accessibility of educational resources is closely related to transportation conditions and the natural environment, and can be featured by road density.

**Table 2:** Pre-selection of factors influencing the level of regional educational development.

Independent variables	Unit	Mean	Standard deviation	Minimum	Maximum
Per capita GDP (*pgdp*)	10^4^Yuan/person	4.0424	2.8332	0.3603	16.4889
Per capita fiscal expenditure on education (*fiscal*)	10^4^Yuan/person	0.1448	0.1091	0.0133	0.7510
Population density (*popden*)	10^4^Person/km^2^	0.0428	0.0607	0.0002	0.3673
Urbanization rate (*urban*)	%	52.70	15.07	20.21	89.60
Road density (*road*)	km/km^2^	0.79	0.50	0.03	2.19

**Fig 1:**
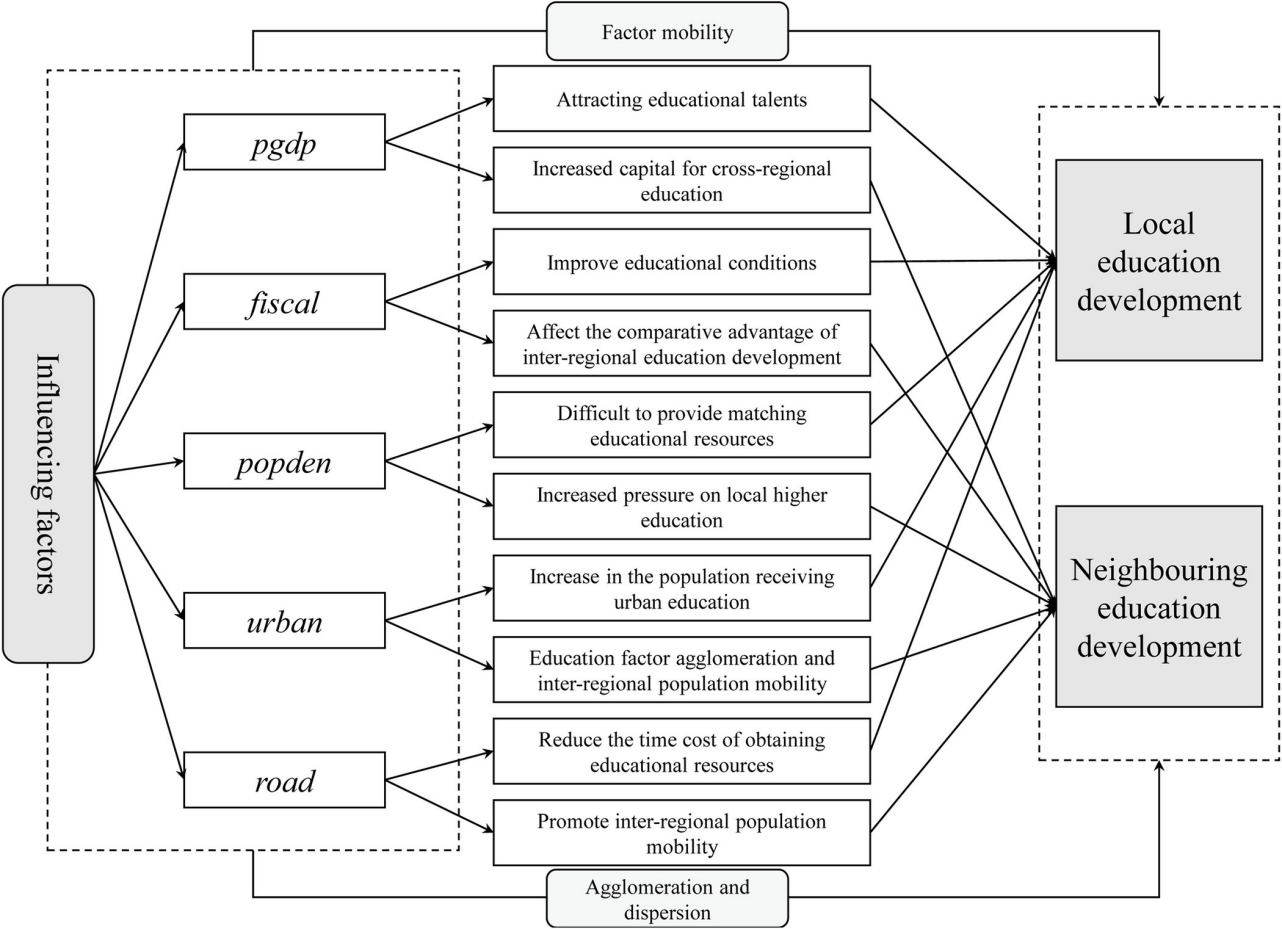
Impact mechanism of influencing factors on EDI.

The impact mechanism of each influencing factor on EDI exists the following paths (Fig 1). The level of regional economic development has two effects, one is to attract high-quality teachers and students, therefore improve local educational development. However, the increase in income also increases the possibility of attending education in neighboring regions, which may have a negative impact on local education development. Investment in education will improve the local education level, but for neighboring areas, when there is a comparative advantage in the construction of educational infrastructure, there will be a siphon effect on education, resulting in the loss of students in surrounding areas, especially high-quality students, which may lead to backward education. A higher population density will increase the local demand for educational resources, and failure to provide adequate educational resources will force students to flow to neighboring areas for education. On the other hand, in areas with high population density, the pressure of study and employment is also great, which often flows to the surrounding areas, and then has an impact on the surrounding areas. Urbanization reflects the difference between urban and rural population in a region. Generally speaking, urban areas have a higher population concentration, capital concentration and talent concentration, and the level of education development is significantly higher than rural areas. Therefore, a higher urbanization rate means that more people can enjoy high-quality urban education, which will contribute to the overall improvement of education levels in the region. Urbanization is an important indicator to measure the economic and social development and progress of a country or region, increasing inter-regional exchanges and thus affecting neighboring regions. Road density reflects the traffic accessibility of an area. When the road density is large, on the one hand, it will affect the time cost of local access to education, and on the other hand, it will promote the smooth exchange of educational talents between regions. The increase in road accessibility also reduces the cost of students from the region to attend education in other areas, which may have an impact on education in neighboring areas.

The results of multicollinearity test show that the Variance Inflation Factors (VIF) of the influencing factors is 3.76, which is less than 10, indicating that there is no high correlation between independent variables, which is suitable for multiple regression analysis.

In traditional econometrics, the research objects are often assumed to be independent. Actually, any phenomenon / thing is spatially correlated [62]. Therefore, spatial econometrics is gaining more and more popularity among scholars. The spatial effects of regional educational development are mainly manifested as follows: the educational development of neighboring provinces is influenced by each other; variables such as per capita GDP of neighboring provinces have an impact on the educational development of the province; other factors not taken into consideration in the province have an impact on the educational development of neighboring provinces. To avoid possible bias in the non-spatial panel model, the spatial panel model is used to analyze the factors influencing the regional inequality in China’s educational development, and the model can be set as follows:

$$\begin{array}{c} Y_{it}=\alpha_{i}+\rho \sum_{k=1}^{N}W_{ik}Y_{kt}+\beta X_{it}+\varphi \sum_{k=1}^{N}W_{ki}X_{kt}+\varepsilon_{it}\\ \varepsilon_{it}=\lambda W\varepsilon_{t}+\nu_{it} \end{array}$$ (8)

where *i* and *k* represent different regions; *W*_*ik*_ is the spatial weight matrix determined according to the Rook’s principle; *X*_*it*_ and *Y*_*it*_ demote the independent variable vector and the dependent variables, respectively; *β* and *φ* are the non-spatial and spatial regression coefficients of the independent variables, respectively; *ρ* is the spatial regression coefficient of the dependent variables; *λ* is the spatial error regression coefficient; *α*_*i*_ is a constant, and *ε*_*it*_ and *v*_*it*_ are the random disturbance term, respectively.

If *ρ* ≠ 0 and *φ* = 0, Formula (8) is a spatially lagged panel data model (SLPDM) that measures the impact of educational development in neighboring provinces on the educational development of the province. If *λ* ≠ 0 and *ρ* = 0, Formula (8) is a spatial error panel data model (SEPDM) that reflects the impact of factors not included in the consideration of a province other than the pre-selected independent variables on the educational development of neighboring provinces. If *ρ* ≠ 0, *φ* ≠ 0 and *λ* = 0, Formula (8) is a spatial Durbin panel data model (SDPDM) that considers the impacts of both the educational development of neighboring provinces and the pre-selected variables of neighboring provinces such as per capita GDP on the educational development of the province.

In the modeling process, the data are first estimated by OLS, and spatial correlation was tested by *LM* test. If there is spatial correlation and the test results support one or both of the spatial lag model and spatial error model, further construction of the spatial Durbin model is required, and the original hypothesis that the spatial Durbin model reduces to a spatial lag model and a spatial error model needs to be tested with the *LR* and *Wald* statistics, respectively. If the test results reject the two original hypotheses, the spatial Durbin model should be chosen. If the former original hypothesis holds and the *LM* test results support the spatial lag model, the spatial lag model is selected. If the latter original hypothesis holds and the *LM* test results support the spatial error model, then the spatial error model is chosen. If the model chosen according to the *LR* test and the *Wald* test is inconsistent with that chosen by the *LM* test, the spatial Dobbin model should also be chosen since it is a general form of spatial lag model and spatial error model. All model analyses are carried out in Stata15.

### 3.3. Data source and processing

The sample used in this study is the 31 provincial administrative units in mainland China from 2003 to 2020, totaling 558. Data on educational opportunities are obtained from the Educational Statistics Yearbook of China (2003–2020); data on educational achievements are collected from the Internet, as well as the China Population Statistics Yearbook (2004–2006) and China Population & Employment Statistics Yearbook (2007–2021); and data on resident population, fiscal expenditure on education, urbanization rate, per capita disposable income and road density were derived from the China Statistical Yearbook (2004–2021). In the statistics on the number of schools, senior secondary schools include combined secondary schools, regular secondary schools and 12-year schools; junior secondary schools include regular junior secondary schools, 9-year schools and vocational junior secondary schools; and external teaching sites are included in primary schools. However, in the statistics of teachers, libraries and other educational resources, they are strictly divided according to primary schools, junior secondary schools and senior secondary schools.

## 4. Results

### 4.1. Measurement of regional inequality in China’s educational development

#### 4.1.1. General trend of China’s educational development

**Fig 2:**
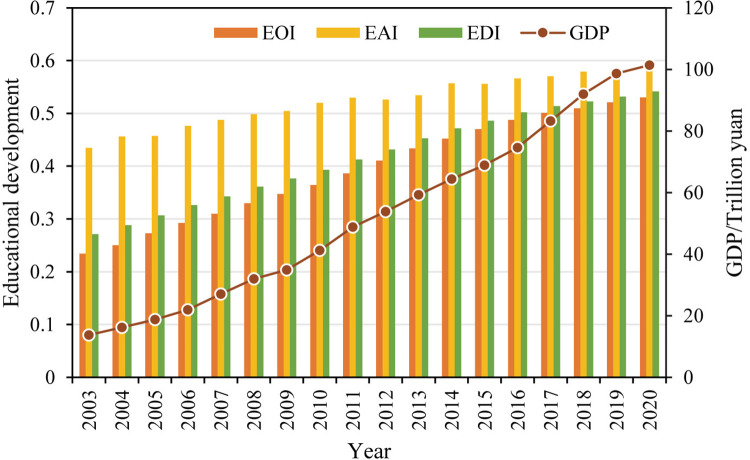
The evolution of China’s educational development from 2003 to 2020.

From 2003 to 2020, China has achieved rapid development in education. The national average of provincial EDI increased from 0.271 to 0.541, with an average annual growth rate (AGR) of 4.15%. The growth rate of the national EDI shows a decreasing trend year by year, from 6.18% in 2003–2004 to 1.75% in 2019–2020 (Fig 2), which indicates that the growth rate of EDI continues to decline as the level of educational development increases. Furthermore, this study analyzes the development of educational opportunities and educational achievement. The results show that China’s educational opportunity index (EOI) and educational achievement index (EAI) increased from 0.234 and 0.435 in 2003 to 0.530 and 0.590 in 2020, with an AGR of 4.92% and 1.81%, respectively. In terms of the trends, EOI shows a similar evolutionary feature as EDI, i.e., the value increases steadily but the growth rate decreases; EAI grows steadily amidst fluctuations, but its growth rate generally shows a downward trend, with negative values in individual years.

#### 4.1.2. China’s educational development at provincial level

**Fig 3:**
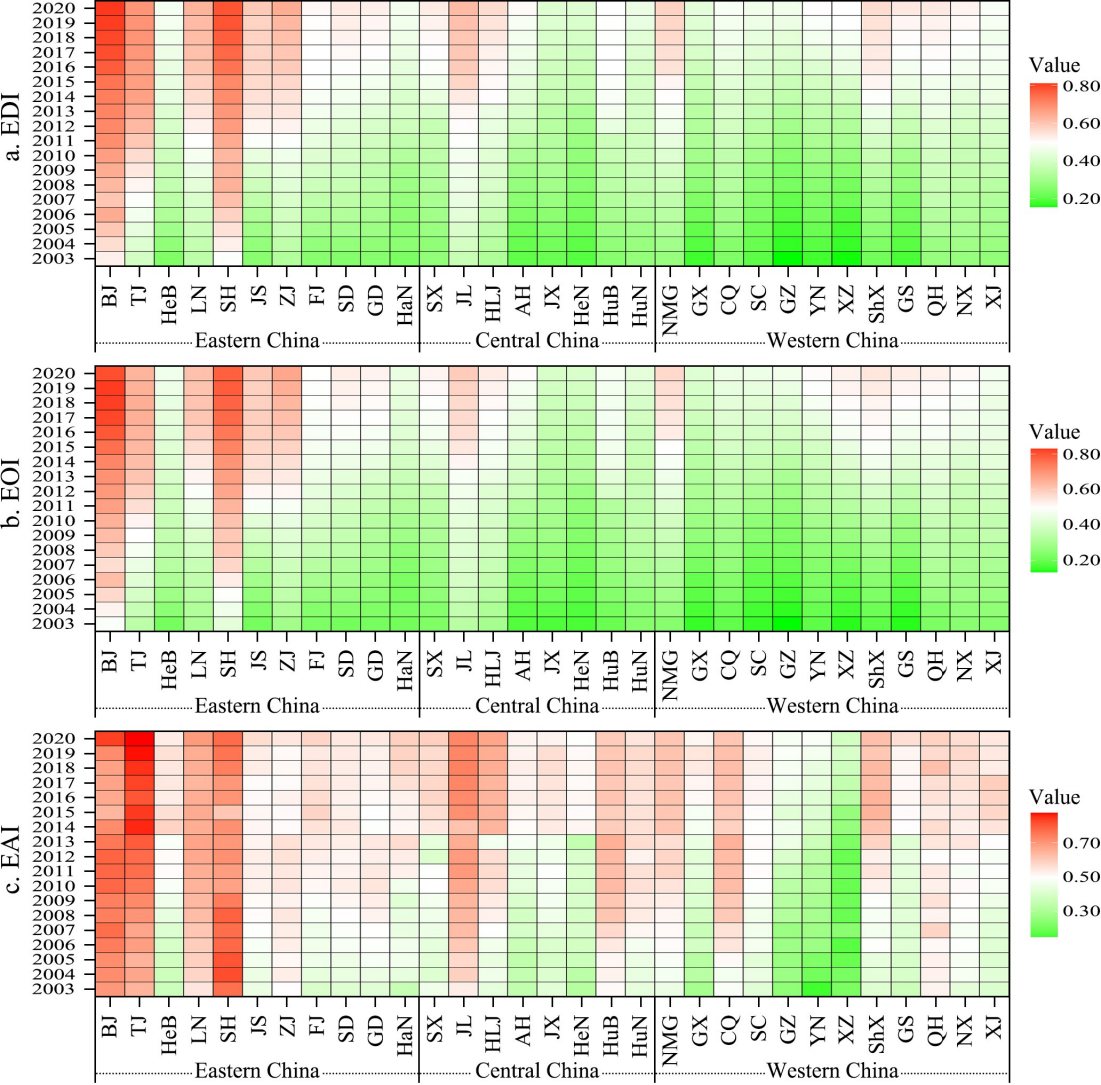
Provincial educational development level in China from 2003 to 2020.

In 2003, the spatial pattern of EDI in China was featured by a low-level of relative balance (Fig 3). 24 provinces had an EDI below 0.30, with Guizhou, Tibet, Guangxi, Yunnan, Gansu and Henan less than 0.20; only Beijing and Shanghai had an EDI above 0.40, at 0.527 and 0.494, respectively. In 2020, 22 provinces had an EDI between 0.45 and 0.60; provinces with EDI below 0.45 were Henan, Jiangxi and Guangxi, with values of 0.421, 0.422 and 0.436, respectively; the provinces with EDI higher than 0.70 were Shanghai and Beijing, with values of 0.782 and 0.813, respectively. Although the regional pattern of EDI has changed considerably in 2003–2020, the gradient pattern of decreasing from eastern to western China is still evident. Specifically, eastern China is an agglomeration of high-value provinces, especially Beijing, Tianjin and the provinces of Yangtze River Delta, which have significantly higher EDI than other provinces; while western China is an agglomeration of low-value provinces. This distribution is consistent with China’s overall level of economic and social development [22]. Therefore, the development of education is affected by the development of regional economy, and comprehensive measures are needed to promote the improvement of education level.

In terms of the EOI and EAI, they both show a clear decreasing feature from eastern to western China and from northern to southern China. In 2003, provinces with high EOI and EAI were mainly distributed in east, northeast and north China; while the low-value provinces were mainly distributed in southwest China. Specifically, the provinces with EOI greater than 0.25 were Zhejiang, Heilongjiang, Liaoning, Jilin, Tianjin, Shanghai and Beijing, with values of 0.290, 0.299, 0.307, 0.335, 0.343, 0.432 and 0.488, respectively; while those with EOI less than 0.15 included Guizhou, Gansu and Tibet, with values of 0.136, 0.146 and 0.146, respectively. Tianjin, Beijing and Shanghai were the only three provinces with EAI greater than 0.60, with values of 0.646, 0.701 and 0.769, respectively; and there were four provinces with EAI less than 0.30, namely Yunnan, Tibet, Guizhou and Guangxi, with values of 0.149, 0.217, 0.249 and 0.291, respectively. In 2020, only Jiangxi had an EOI less than 0.40, and the value was 0.399; the provinces with EOI greater than 0.60 were Jiangsu, Liaoning, Tianjin, Zhejiang, Shanghai and Beijing, and the values were 0.602, 0.619, 0.642, 0.667, 0.784 and 0.808, respectively. There were four provinces with EAI less than 0.50, namely Tibet, Guizhou, Yunnan and Henan, with values of 0.386, 0.478, 0.485 and 0.493, respectively; the number of provinces with EAI greater than 0.70 was also four, namely Jilin, Shanghai, Beijing and Tianjin, and their values were 0.730, 0.775, 0.836 and 0.876, respectively. The provinces with low EOI and EAI levels are concentrated in the western region of China, which is the most underdeveloped region in the country. These provinces have complex terrain, low traffic accessibility and lagging economic and social development, so the construction of educational facilities, teacher quality and other insufficient development, restricting the level of regional education development [41]. It is worth noting that Henan Province has a low EAI value in 2020, but its GDP ranks fifth in the country, and its low EAI value is mainly related to its large population size and lack of higher education resources.

#### 4.1.3. Regional inequality of China’s educational development

**Fig 4:**
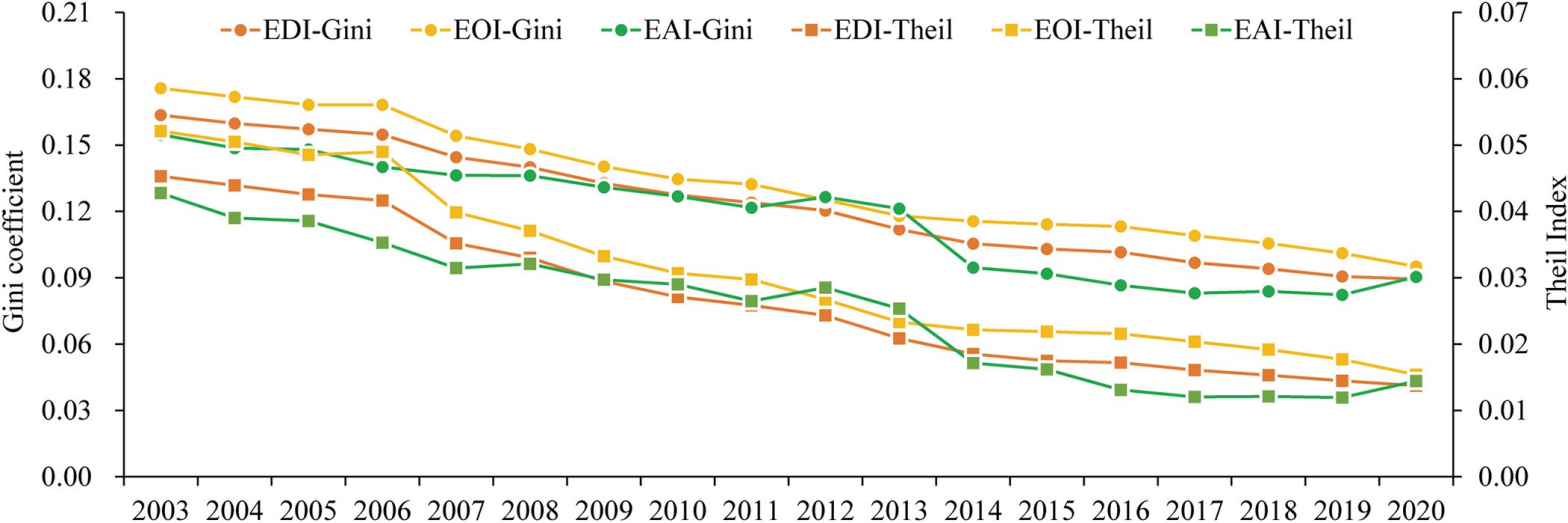
Regional inequality in China’s educational development from 2003 to 2020.

Here, the ratio of maximum to minimum values is calculated to reveal the dispersion of educational development levels. The continuous decrease in this ratio indicates that the fluctuations in the provincial educational development level are decreasing. Specifically, the ratio of the maximum to minimum provincial EDI decreases from 3.37 in 2003 to 1.93 in 2020, and the values of EOI and EAI respectively decreases from 3.60 and 5.16 to 2.02 and 2.24 over the same period. Additionally, the Gini coefficient and Theil index of education development levels are calculated to analyze the degree of regional inequality in China’s educational development. The Gini coefficients of provincial EDI, EOI and EAI decreased from 0.1636, 0.1758 and 0.1547 in 2003 to 0.0895, 0.0951 and 0.0904 in 2020, respectively; and the Theil index dropped from 0.0453, 0.0521, and 0.0428 to 0.0137, 0.0154 and 0.0144, respectively (Fig 4). With the rapid growth of education levels in backward regions, the education gap between different provinces in China has gradually narrowed, as evidenced by the continued decline in the Gini coefficient and Thiel index. This suggests that China has made remarkable achievements in promoting balanced educational development.

**Fig 5:**
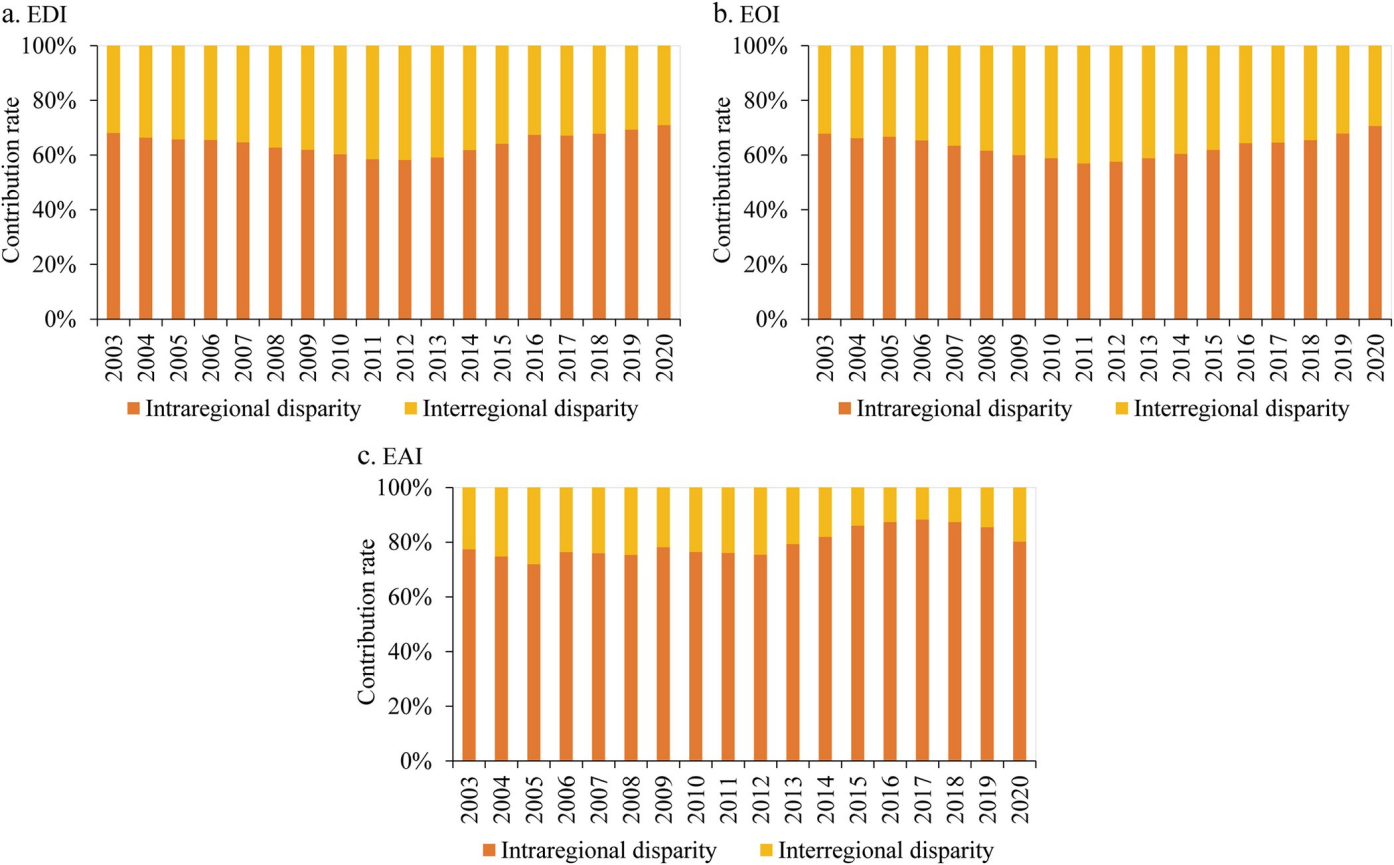
Decomposition of the regional disparity in China’s educational development from 2003 to 2020.

According to the geographical division of eastern, central and western China, this study decomposes the Theil index of China’s educational development level and analyzes the contribution rate of interregional disparities and intraregional disparities to national regional disparities (Fig 5). In 2003–2020, the contribution rate of intraregional disparities generally gains a small increase. The contribution rates of intraregional disparities of EDI, EOI and EAI increase from 68.08%, 67.82% and 77.37% to 70.96%, 70.62% and 80.18%, respectively, indicating that the regional disparities in China’s educational development mainly originate from intraregional disparities, with less contribution from interregional disparities. The intraregional disparities of both EDI and EOI show an evolutionary trend of decreasing and then increasing, with the lowest values being 58.23% in 2012 and 56.91% in 2011, respectively, and the highest values appearing in 2020; the intraregional disparities of EAI show a fluctuating increasing trend, with the lowest and highest values being 71.93% in 2005 and 88.26% in 2017, respectively. These evolutionary trends suggest that since the second decade of the 21st century, the interregional disparities of EDI and EOI have narrowed, but the intraregional disparities continue to widen; the intraregional disparity of EAI begin to decline after the 19th National Congress of the Communist Party of China, while the interregional disparity continues to widen. These results suggest that despite significant interregional disparity in education levels between eastern, central, and western regions, intraregional disparity between provinces is still dominant, which should be addressed by the Chinese government in the future.

### 4.2. Driving mechanism of China’s education inequity

#### 4.2.1. Model selection

Spatial econometric analysis presupposes the existence of spatial dependence [63]. Thus, Moran’s *I*, an important index in exploratory spatial data analysis, is employed to examine the spatial auto-correlation of China’s educational development. If spatial autocorrelation exists, a spatial econometric model should be chosen to analyze the data. Spatial autocorrelation refers to the phenomenon that the high value tends to cluster with the high value and the low value tends to cluster with the low value. For the calculation of Moran’s *I*, please refer to [64]. In general, the value of Moran’s *I* is between -1 and 1. The closer to 1 indicates a stronger spatial positive correlation of provincial educational development; the closer to -1 indicates a stronger spatial negative correlation of provincial educational development; and a value of 0 indicates that no spatial autocorrelation of provincial educational development, i.e., spatially random distribution. As can be seen from Table 3, the Moran’s *I* of China’s provincial EDI from 2003 to 2020 are all greater than 0 and pass the significance level test of 1%, indicating that there is a significant positive autocorrelation among China’s EDI, i.e., provinces with similar EDIs show a characteristic of spatial clustering. Meanwhile, the Moran’s *I* of EOI and EAI in 2003–2020 also indicate that the provinces with similar EOI and EAI show a clustered distribution in space.

**Table 3:** Moran’s *I* of China’s educational development from 2003 to 2020.

Year	Moran’s *I*			Year	Moran’s *I*		
	EDI	EOI	EAI		EDI	EOI	EAI
2003	0.263***	0.277***	0.202***	2012	0.320***	0.332***	0.250***
2004	0.282***	0.303***	0.189***	2013	0.301***	0.339***	0.153***
2005	0.282***	0.301***	0.191***	2014	0.327***	0.317***	0.270***
2006	0.280***	0.302***	0.168***	2015	0.320***	0.299***	0.276***
2007	0.295***	0.326***	0.167***	2016	0.295***	0.281***	0.250***
2008	0.304***	0.332***	0.194***	2017	0.280***	0.265***	0.225***
2009	0.316***	0.346***	0.182***	2018	0.268***	0.251***	0.206***
2010	0.326***	0.350***	0.217***	2019	0.259***	0.238***	0.236***
2011	0.341***	0.361***	0.243***	2020	0.258***	0.244***	0.261***

Note:

*, ** and *** denote *p*<0.1、*p*<0.05 and *p*<0.01, respectively; and the same applies to the following tables.

It can be seen from the above analysis that China’s educational development has a significant spatial correlation. Therefore, it is necessary to employ spatial panel econometric to investigate the factors influencing regional inequality in China’s educational development. According to Elhorst (2014), the results of the *LM* test, *LR* test and *Wald* test suggest that SDPDM should be selected to diagnose the influencing factors of China’s EDI [65]. The results of *Hausman* test indicate that the fixed effect model should be chosen. Then we use *LR* test to diagnose the time-period fixed effects model, spatial fixed effects model and two-way fixed effects model, and the results suggest that the two-way fixed effects model should be selected (Table 4).

**Table 4:** Results of *LM* test, *LR* test, *Wald* test and *Hausman* test.

		EDI	
*LM* test		Spatial error	Spatial lag
	Lagrange multiplier	31.418***	31.508***
	Robust Lagrange multiplier	23.097***	23.187***
*LR* test			
	Comparison of SDM and SAR	39.91***	
	Comparison of SDM and SER	40.98***	
*Wald* test		chi2(5)	
	For spatial lag	41.07****	
	For spatial error	42.15***	
*Hausman* test	Comparison of FE and RE	160.04***	
*LR* test	Comparison of both and ind	187.04***	
	Comparison of both and time	675.42***	

#### 4.2.2. Model estimation results

Economic development is the result of resource allocation, and the level of regional economic development also determines the regional ability to allocate resources [66]. In general, a high per capita GDP (*pgdp*) means a high level of regional economic development. In this context, the influx of well-educated people from neighboring areas, driven by economic interests and household needs, will increase the proportion of people with junior secondary school education and above in local total population. Meanwhile, a high level of regional economic development means that it has a greater ability to support a higher-level education for its school-age population. However, the estimation results shows that *pgdp* has a positive direct effect, but it fails passing the significance test (Table 5). On the other hand, the higher level of economic development in the surrounding areas will also attract the inflow of local teachers and other educational resources, therefore, we recognize a significant negative indirect spillover effect on the development of local EDI.

**Table 5:** Estimation results of spatial Durbin panel data model.

Variables	Main	Direct effect	Indirect	Total
			effect	effect
pgdp	0.0006	0.0007	-0.0052***	-0.0045**
	(0.0012)	(0.0013)	(0.0017)	(0.0019)
fiscal	0.1570***	0.1580***	-0.1300***	0.0280
	(0.0232)	(0.0226)	(0.0404)	(0.0434)
popden	0.1171	0.1003	1.8774***	1.9778***
	(0.1539)	(0.1464)	(0.3577)	(0.3653)
urban	0.1718***	0.1685***	0.3186***	0.4871***
	(0.0497)	(0.0498)	(0.0685)	(0.0732)
road	-0.0125	-0.0123	-0.0182	-0.0305***
	(0.0085)	(0.0083)	(0.0143)	(0.0118)
Observations	558	558	558	558
R^2^	0.4811	0.4811	0.4811	0.4811

Note: Standard errors in parentheses.

Local per capita fiscal expenditure on education (*fiscal*) has a significant positive direct effect on educational development, while *fiscal* in neighboring areas presents a negative effect on local educational development. Fiscal expenditure on education is the material basis for regional educational development. The increase of per capita fiscal expenditure on education promotes the development of regional education by increasing the supply of regional educational resources. However, the improvement of educational opportunities resulting from the increase of per capita financial expenditure on education in neighboring areas reduces the attractiveness of the local area to the population of the surrounding areas. On the other hand, this will attract local population to the neighboring areas to a certain extent, which has a significant negative indirect spillover effect on local EDI.

In terms of the population density, only the population density of neighboring areas has a significant positive indirect spillover effect on local EDI. This is mainly because high population density means more competition among people, promoting the migration of many well-educated people to neighboring areas and boosting local EDI.

Urbanization is essentially a process of factor agglomeration [67, 68]. The increase in urbanization rate helps to centralize the allocation of educational resources and enhance the level of local educational development. At the same time, the importance of education in urban civilization requires that people receive a good education and improve their quality to meet the needs of industrialization. On the other hand, the increased level of industrialization brought about by the urbanization of the neighboring areas has created an attraction for local population, which increases local supply of educational opportunities and raises local level of educational development. Therefore, urbanization has a significant direct and indirect positive spillover effects.

Local road density has a significant inhibitory effect on regional educational development in terms of the total spatial spillover effect. This means that a one-unit increase in the density of the road network across the region reduces the local EDI by 0.0305 units. This is mainly due to the fact that the increase in road density increases the availability of educational resources and a given number of educational resources can be accessed by more people. Meanwhile, increased road density allows for a smoother exchange of talent between regions, thereby reducing local EDI.

#### 4.2.3. Regional heterogeneity analysis

In this study, China was divided into three regions: eastern, central and western regions, and the heterogeneity of the model was analyzed (Table 6). The per capita GDP has a significant positive direct spillover effect in the eastern region and a significant negative indirect spillover effect in the central region. In the western region, per capita GDP presents a significant negative direct spillover effect and a positive indirect spillover effect. On the whole, per capita GDP in the western region presents a total positive spillover effect.

**Table 6:** Estimation results of spatial Durbin panel data model by regions.

	Eastern region				Central region				Western region			
	Main	Direct effect	Indirect effect	Total effect	Main	Direct effect	Indirect effect	Total effect	Main	Direct effect	Indirect effect	Total effect
pgdp	0.0094***	0.0095***	0.0003	0.0097***	0.0032	0.0065	-0.0519**	-0.0454	-0.0024	-0.0090***	0.0274***	0.0184***
	(0.0017)	(0.0018)	(0.0018)	(0.0020)	(0.0090)	(0.0085)	(0.0244)	(0.0302)	(0.0019)	(0.0024)	(0.0038)	(0.0028)
fiscal	-0.1612**	-0.1279*	-0.3166***	-0.4446***	-0.0271	0.0004	-0.6573	-0.6569	0.0918***	0.1261***	-0.1454***	-0.0194
	(0.0724)	(0.0685)	(0.0751)	(0.1019)	(0.2381)	(0.2181)	(0.4703)	(0.6284)	(0.0216)	(0.0226)	(0.0344)	(0.0304)
popden	0.4364***	0.3436**	0.9220***	1.2656***	-0.4720	-0.5092	2.3163	1.8070	-1.1599	-1.5158*	1.8102	0.2944
	(0.1537)	(0.1411)	(0.2673)	(0.2897)	(1.3536)	(1.2575)	(1.5314)	(2.3519)	(0.7738)	(0.7986)	(1.5150)	(1.4516)
urban	0.1122*	0.0717	0.3670***	0.4387***	0.7604**	0.7309**	0.5869	1.3178*	-0.0564	0.0426	-0.4221***	-0.3795***
	(0.0611)	(0.0656)	(0.0632)	(0.0843)	(0.3180)	(0.2844)	(0.5879)	(0.7994)	(0.0879)	(0.0915)	(0.1301)	(0.1344)
road	-0.0230*	-0.0367**	0.1118***	0.0750***	0.0314	0.0329	-0.0268	0.0061	-0.0418***	-0.0688***	0.1088***	0.0401***
	(0.0125)	(0.0159)	(0.0209)	(0.0149)	(0.0227)	(0.0206)	(0.0388)	(0.0514)	(0.0092)	(0.0125)	(0.0216)	(0.0148)
Observations	234	234	234	234	108	108	108	108	216	216	216	216
R^2^	0.3055	0.3055	0.3055	0.3055	0.0062	0.0062	0.0062	0.0062	0.0056	0.0056	0.0056	0.0056

Note: Standard errors in parentheses.

The *fiscal* exhibits total negative spillover effect in the eastern region, indicating that when the *fiscal* increase 1 unit, the local educational level will decrease by 0.4446 units. In the central region, the effect of *fiscal* is not significant. In the western region, *fiscal* presents a significant positive direct effect, indicating that 1 unit of *fiscal* increase in the region will increase the local education level by 0.1261 units. However, *fiscal* also presents a significant negative indirect effect, indicating that 1 unit of fiscal increase in neighboring regions will decrease local educational level by 0.1454 units.

*Popden* has significant positive direct and indirect spillover effects in the eastern region, indicating that the increase of population density in the eastern region is conducive to the improvement of the overall regional education level. The effect is not significant in the central region. In the western region, *popden* shows only a significant direct negative effect, indicating that an increase in population density leads to a decrease of 1.5158 units in the level of education. This may be related to the lack of educational resources in the western region.

*Urban* has a significant positive indirect spillover effect in the eastern region, and its total effect is positive, indicating that the improvement of urbanization level in all provinces in this region is conducive to the improvement of local education level. In the central region, the total effect of urbanization is positive, indicating that for every 1 unit increase in the urbanization rate of all provinces in the central region, the local education level increases by 1.3178 units. In the western region, urbanization presents a significant negative indirect effect, and the total effect is negative, indicating that for every 1 unit increase in the urbanization level in all provinces in the western region, the local education level will decrease by 0.3795 units.

*Road* fails passing the significance test in the central region. In the eastern and western regions, road density has a significant negative direct effect and a significant positive indirect effect, and the total effect is positive. This indicates that the improvement of road level will reduce the local educational level, but the improvement of road density in the neighboring provinces will improve the local educational level. From the total effect, the improvement of road density in all provinces will improve the local educational level.

### 4.3. Robustness test

We choose the inverse distance square as a new weight matrix to calculate the model. In addition, we also choose to exclude Beijing and Shanghai, the two regions with the highest level of educational performance, to test the robustness of the model (Table 7). The results show that *fiscal* and *urban* still have significant positive effects. In the total spatial spillover effect, when the weight matrix is replaced, *pgdp* and *road* show a significant negative spillover effect, while *popden* and *urban* exhibit a significant positive spillover effect, which is consistent with the results of the main model. However, after excluding Beijing and Shanghai, *popden* shows a significant negative effect, which is contrary to the results of the main model, but the regression results of other factors are consistent. On the whole, the model regression results have strong robustness.

**Table 7:** The results of robust tests.

	Replacement weight matrix				Excluding Beijing and Shanghai			
	Main	Direct	Indirect	Total	Main	Direct	Indirect	Total
		effect	effect	effect		effect	effect	effect
pgdp	-0.0019	-0.0017	-0.0091***	-0.0108***	0.0033**	0.0034**	-0.0019	0.0015
	(0.0012)	(0.0012)	(0.0031)	(0.0032)	(0.0015)	(0.0015)	(0.0024)	(0.0023)
fiscal	0.1722***	0.1782***	-0.4025***	-0.2244**	0.1867***	0.1838***	-0.2003***	-0.0165
	(0.0236)	(0.0239)	(0.0954)	(0.0949)	(0.0231)	(0.0226)	(0.0481)	(0.0526)
popden	0.4417***	0.4058***	2.9001***	3.3059***	0.1918	0.1843	-3.8035***	-3.6192***
	(0.1524)	(0.1479)	(0.4383)	(0.4812)	(0.3329)	(0.3217)	(1.0625)	(1.0179)
urban	0.2703***	0.2695***	0.2184*	0.4879***	0.1875***	0.1917***	0.3159***	0.5076***
	(0.0505)	(0.0512)	(0.1288)	(0.1213)	(0.0558)	(0.0556)	(0.0850)	(0.0922)
road	-0.0120*	-0.0114*	-0.0388	-0.0502**	-0.0340***	-0.0339***	0.0271*	-0.0068
	(0.0070)	(0.0069)	(0.0263)	(0.0252)	(0.0087)	(0.0084)	(0.0157)	(0.0133)
Observations	558	558	558	558	522	522	522	522
R^2^	0.2237	0.2237	0.2237	0.2237	0.3941	0.3941	0.3941	0.3941

Note: Standard errors in parentheses.

## 5. Discussion

### 5.1. Suggestions for China’s educational development

In the past few decades, the accelerated economic globalization and increasingly fierce international competition have posed enormous challenges to developing countries [69]. In essence, international competition is a comprehensive national power competition based on economic and scientific strength, the key to which lies in talent [70]. Therefore, countries and regions around the world attach great importance to educational development and strive to provide quality education for their children and youth to achieve the conversion of human resources to human capital. Meanwhile, the United Nations (UN) has incorporated education as an important content in action plans to guide global development, such as MDGs and SDGs, and has launched global initiatives such as the Global Education First Initiative (GEFI) and the Futures of Education to mobilize broad international supports for education. A comprehensive review of the 17 goals outlined in the 2030 Agenda for Sustainable Development suggests that education is the key to achieving the other goals [71, 72] and that access to quality education is fundamental to improving livelihood and achieving sustainable development.

As the world’s largest developing country, China has long been actively developing education to improve the quality of its people and turn its demographic advantage into a human capital advantage [9, 73]. From large-scale literacy campaigns to universal nine-year compulsory education, and then to the leapfrogging of higher education, China has gradually established a modern educational system. However, regional inequalities in China’s educational development are extremely significant and will persist for a long time due to the *hukou* division given by administrative divisions and the spatial heterogeneity of location. Meanwhile, regional inequality in educational development has become a key factor affecting China’s economic inequality since the important role of education in human capital formation [22, 74, 75]. After more than 70 years of development, China’s economic development has now entered a critical period of transformation [76, 77]. Adapting to the new normal of economic development, active development of education has become an important driving force to help shift the economic growth momentum from factor-driven and investment-driven to innovation-driven. Therefore, some targeted measures need to be taken to narrow the educational development gap between regions and help the high-quality development of China’s economy.

First, the government should further strengthen the optimal allocation of factors to promote regional economic development to a higher level since the regional economy is the basis for educational development. Meanwhile, the shortcomings of economic development in the backward areas needs to be filled to build a new pattern of high-quality regional economic development. The results of heterogeneity analysis show that the improvement of economic development level can significantly improve regional education level in eastern and western regions. The eastern region should give full play to its scientific and technological advantages, promote industrial upgrading through innovation, and improve the efficiency of economic development. The western region should rely on the beautiful ecological environment and resources and industries with regional characteristics to develop the ecological economy and improve the resilience of economic development. Second, the government should make education the focus of livelihood protection and increase fiscal investment in education to increase the supply of educational opportunities; at the same time, the government should optimize the fiscal expenditure structure and narrow the gap in regional educational development by strengthening policy support for backward areas such as the central and western regions. Especially in the western region, the improvement of fiscal can significantly promote the development of education in the region, so the proportion of education expenditure should be increased, and the central government should increase the transfer payment to ensure the continuous growth of education investment in the western region, so as to reduce the inequality of regional education development. Third, special attention should be paid to increasing the supply of educational resources in densely populated areas, ensuring equal opportunities for each person to obtain educational services, and promoting balanced regional education development. Fourth, there is an urgent need to promote people-oriented urbanization and improve the level and quality of urbanization, raising the utilization efficiency of educational resources and the level of educational development by bringing into play the scale effect brought by the agglomeration of educational resources. The construction of urbanization will help promote the development of education in the eastern and central regions. The eastern region should focus on improving the quality of urbanization and promoting sustainable and inclusive urbanization. The central region should continue to improve the construction of infrastructure and public services in urban areas, attract rural people to work in urban areas, and promote the further improvement of the level of urbanization. Finally, on the basis of improving regional accessibility, the local government should strengthen the transportation links with the neighboring areas to create conditions for the optimal allocation of interregional educational resources and the free migration of the well-educated population. Road facilities have a significant positive effect on the eastern and western regions as a whole. The eastern region should improve road connectivity and build a road network commensurate with the level of economic development, while the western region should focus on increasing road construction in hilly, mountainous and remote areas to further expand the coverage of the road network.

As a member of the Global South, China has built the world’s largest education system on the back of its rapid economic growth. The Chinese government has guaranteed the people’s right to education through subsidies and legislation. At the same time, the social atmosphere that the whole people attach great importance to education has greatly promoted the development of education in China. In the other countries of the Global South, the lagging development of education remains an obstacle to comprehensive socioeconomic transformation. There is a need for extensive cooperation, economic support and enhanced role in global knowledge networks to continuously upgrade education in the Global South.

### 5.2. Limitations and future prospects

With the acceleration of globalization, marketization, industrialization and urbanization as well as changes in migration policies, the size of China’s migrant population has increased rapidly over the past four decades [78]. From 1982 to 2000, the total migrant population increased from 6.57 million to 121 million, and the proportion of the total population increased from 0.6% to 9.6%; by 2020, the total migrant population increased to 376 million, with a proportion of 26.6%. It can be said that China has entered an era of migration and has reached a high level [79]. However, the existence of the *hukou* system prevents migrant populations from having equal access to local public services such as education, resulting in social separation [80–82]. Therefore, more attention should be paid to ensuring that the migrant population can enjoy public services equally to promote educational equity. In terms of migration direction, rural-to-urban migration has been dominant, leading to the hollowing of rural educational facilities [83, 84]. In contrast, problems such as large class sizes and large school sizes are common in cities [85, 86]. In the case of limited regional fiscal resources, how to coordinate factor inputs to realize optimal allocation of urban and rural educational resources deserves further research. According to the Ministry of Education of the PRC, the enrollment rate of China’s compulsory education was close to 100% in 2020, and more than 95% of counties had passed the assessment and recognition of basic balanced development of compulsory education [87]. When the education coverage reaches a certain level, regional inequality of educational development will change from quantitative gaps to qualitative disparities [88]. Thus, national policies on educational development should focus on the supply of high-quality education and the improvement of educational achievements when the quantitative shortage of educational resources has been effectively addressed.

To investigate the characteristics of inequality in regional educational development, this study constructs a comprehensive evaluation index system from two dimensions of educational opportunity and educational achievement. However, the availability of data made the selection of specific indicators focus on the dimension of educational opportunities and reveal mainly the number of educational opportunities. Insufficient attention has been paid to indicators characterizing the quality of educational opportunities, such as teaching skills and the utilization of educational facilities. Meanwhile, only four indicators were selected to reflect educational achievement, with a lack of systematic consideration on educational achievements such as the competitiveness and ability of graduates. Especially at a time when people are paying more and more attention to education, educational equity, as an important content of educational achievement, deserves to be explored in depth. According to the Education Law of the PRC, China implements the school education system of preschool education, primary education, secondary education and higher education. However, only primary and secondary education are included in this study, resulting in an inevitable bias in the understanding of regional education development.

## 6. Conclusions

China is a vast country with diverse topography [89]. The natural and human differences between regions determine the existence of significant regional inequalities in China’s educational development. This study explores the connotation of educational development, constructs an evaluation index system, including 2 dimensions, 5 subdimensions and 26 specific indicators, to measure the level of regional educational development, and then investigates the characteristics of regional inequality in China’s educational development and its influencing factors. From 2003 to 2020, China’s EDI, as well as EOI and EAI, grow steadily, but the evolution of their growth rates shows a downward trend. Spatially, EDI maintains a gradient decreasing pattern from eastern China to western China, and EOI and EAI show a decreasing pattern from the east to the west and from the north to the south. The decreasing Gini coefficient and Thiel index indicates that the regional disparity in China’s educational development continues to narrow, and the decomposition of the Thiel index suggests that the regional disparity stems primarily from the intraregional inequalities. In terms of direct spillover effects, per capita fiscal expenditure on education and urbanization rate have positive effects. In terms of indirect spillover effects, per capita GDP and per capita fiscal expenditure on education have negative effects, while population density and urbanization rate have positive effects. In line with needs of socio-economic transformation in the new era, it is necessary to build a new pattern of high-quality regional economic development, strengthen fiscal investment in education in backward areas, actively promote people-oriented urbanization as well as improve intra- and inter-regional transportation conditions, thus giving full play to the important role of education in regional development. In addition, regional heterogeneity analysis provides new insights different from global regression analysis, so it is necessary to formulate differentiated policy measures according to the driving mechanism of impact factors in different regions to improve the efficiency of policy measures.
